# Enzyme activities during Benzo[a]pyrene degradation by the fungus *Lasiodiplodia theobromae* isolated from a polluted soil

**DOI:** 10.1038/s41598-020-57692-6

**Published:** 2020-01-21

**Authors:** Huimin Cao, Cuiping Wang, Haibin Liu, Weili Jia, Hongwen Sun

**Affiliations:** MOE Key Laboratory of Pollution Processes and Environmental Criteria, College of Environmental Science and Engineering, Nankai University, Tianjin, 300071 People’s Republic of China

**Keywords:** Enzymes, Pollution remediation

## Abstract

The enzyme activities of the fungus *Lasiodiplodia theobromae* (*L. theobromae*) were studied during degradation of benzo[a]pyrene (BaP). The *L. theobromae* was isolated from a polycyclic aromatic hydrocarbons (PAHs) contaminated soil collected from the Beijing Coking Plant in China and can potentially use BaP as its sole carbon source with a degradation ratio of up to 53% over 10 days. The activities of lignin peroxidase (LiP) and laccase (LAC) could be detected during BaP biodegradation; while manganese peroxidase (MnP) was not detected. Both glucose and salicylic acid enhanced BaP biodegradation slightly. In contrast, the coexistence of phenanthrene (PHE) inhibited BaP degradation. These metabolic substrates all enhanced the secretion of LiP and LAC. The addition of Tween 80 (TW-80) enhanced BaP biodegradation as well as the LiP and LAC activities. At the same time, TW-80 was degraded by the *L. theobromae*. In addition, the *L. theobromae* was compared to *Phanerochaete chrysosporium (P. chrysosporium)*, which is a widely studied fungus for degrading PAH. *P. chrysosporium* was unable to use BaP as its sole carbon source. The activities of LiP and LAC produced by the *P. chrysosporium* were less than those of the *L. theobromae*. Additionally, the four intermediates formed in the BaP biodegradation process were monitored using GC-MS analysis. Four metabolite concentrations first increased and then decreased or obtained the platform with prolonged BaP biodegradation time. Therefore, this study shows that the *L. theobromae* may be explored as a new strain for removing PAHs from the environment.

## Introduction

Polycyclic aromatic hydrocarbons (PAHs) are a class of diverse organic compounds containing two or more fused aromatic rings of carbon and hydrogen atoms, which can be easily adsorbed into soil due to their highly hydrophobic, stability and strong recalcitrant nature, and its toxicity, mutagenicity and carcinogenicity present a significant threat to human health^1^. PAHs with more than four rings are highly recalcitrant and resistant to microbial degradation^2^. Benzo[a]pyrene (BaP) is a PAH that consists of five fused benzene rings and is one of the most potent carcinogenic PAHs. It has a very low water solubility, vapor pressure, and a high octanol/water partitioning coefficient, suggesting a preference for non-aqueous phases and low microbial bioavailability. Therefore, great efforts have been made to develop strategies for BaP biodegradation.

Due to the recalcitrance of BaP, most studies on BaP biodegradation have focused on the co-metabolization of BaP in the presence of one or more alternative carbon sources^3^. For examples, *Sphingomonas paucumobilis* EPA505 removed 28% of BaP within 2 d when induced by fluoranthene^4^, and *S. yanoikuyae* JAR02 mineralized 3.8% of radio-labeled BaP with the addition of salicylate^5^. Kanaly and Harayama^6^ reported that a bacterial consortium could degrade between 33% and 65% of BaP within 16 d of cultivation in the presence of diesel fuel. Tiwari *et al*.^7^ noted that 34–37% degradation of BaP, by Stenotrophomonas maltophilia strain VUN 10,003 after 42 days of incubation in the presence of phenanthrene and pyrene. However, few studies have isolated microorganisms using BaP as their sole carbon and energy source. Wu *et al*.^8^ reported that *Ochrobactrum sp*., which was isolated from marine sediments in the Western Sea in Xiamen, China, can use BaP as the sole carbon source and degrades approximately 20% of BaP after 30 d of incubation. Rafin *et al*.^9^ obtained a fungal strain of *Fusarium solani* that degraded only 8% of BaP after 12 d of incubation. Thus, the isolation of a novel species that can efficiently use BaP as its sole carbon source is still impending.

Recent studies have focused on the degradation of BaP by white-rot fungi^10,11^. White rot fungi like *Phanerochaete chrysosporium*, *Trametes versicolor*, *Cirnipellis stipitaria* and *Pleurotus ostreatus* have the ability to efficiently degrade most PAHs as solely carbon source. Enzymatic mechanisms for PAH degradation by white-rot fungi have been widely discussed, and it has been recognized that white-rot fungi degrade PAHs by the synthesis of lignin modifying enzymes like lignin peroxidises (LiP), manganese peroxidases (MnP), laccases (LAC) and other oxidases^12^. These enzymes usually catalyze the first attack on the PAHs molecule^2^. It was reported that LiP and MnP can directly catalyze the one-electron oxidation of PAHs with an ionization potential (IP) of up to 7.55 eV to produce PAH quinones^13,14^, which can be further metabolized via ring fission^15^. In addition, LAC can also catalyze the one-electron oxidation of PAHs, such as anthracene (ANT) and BaP. The efficiency of LAC is enhanced in the presence of mediators, such as 1-hydroxybenzotriabole (HBT) or 2, 2’-azinobis-3-ethylbenzthiazoline-6-sulfonic acid (ABTS)^16^. Except these well studied species, Hadibarata and Kristanti^10^ recently noted that LAC and 1, 2-dioxygenase were produced by *Polyporus sp*. S133, a white-rot fungus isolated from an oil-contaminated soil, which might play an important role in the transformation of BaP.

A novel fungus that can use BaP as sole carbon was isolated from a PAH contaminated soil sample in the Beijing Coking Plant in Beijing, China by our research group, which was identified as the *Lasiodiplodia theobromae* (*L. theobromae*)^17^. This fungu belongs to the group of botryosphaeriaceous fungi, is a causal agent of storage rot in many fruits and tubers and is a serious pathogen for many agricultural and horticultural crops^18^. Ligninolytic enzymes are responsible for the wood cankers that are caused by this group of fungi^19^. The ability to secret ligninolytic enzymes is potentially essential for white rot fungi, which is a topic of interest in PAH degradation studies^20^. Thus, it is important to assess the enzyme mechanisms that are involved in the degradation of BaP by the *L. theobromae*. The purpose of this study was to elucidate the enzymatic mechanisms of BaP degradation by the *L. theobromae*. To do so, BaP was used as the sole carbon and energy source for the *L. theobromae* and ligninolytic enzymes produced during the BaP biodegradation were assessed. Furthermore, the effects of several metabolic substrates, glucose, salicylic acid, phenanthrene (PHE), and the Tween-80 surfactant on BaP degradation by the *L. theobromae* and enzyme activities were discussed. Finally, to confirm the potential of the *L. theobromae* as an efficient BaP degrader, a comparative study of BaP degradation and enzymatic activities was conducted between the *L. theobromae* and a well-known PAH-degrading fungus, *P. chrysosporium*.

## Materials and Methods

### Chemicals, media and microorganisms

BaP and PHE (purities >98%) were purchased from the Aldrich Chemical Company (Milwaukee, WI, USA). Stock solutions of PHE and BaP (1 g L^−1^) were prepared in high performance liquid chromatography (HPLC)-grade acetone, respectively. The Tween-80 surfactant (polyoxyethylenesorbitan monooleate, TW-80) was purchased from Sigma Chemicals (St Louis, MO, USA). HPLC grade ethyl acetate and methanol were purchased from Baker Company (Deventer, the Netherlands). All other reagents were of analytic grade.

Mineral salts media (MSM) was prepared and used following the methods of Tao *et al*.^21^. All chemicals that were used in preparing the media were of analytical grade. The liquid media was autoclaved at 121 °C for 30 min. Beef extract-peptone and potato-dextrose media were prepared by using standard methods.

The *L. theobromae* was isolated from a PAH-contaminated soil sample at the Beijing Coking Plant in Beijing, China. *P. chrysosporium* was purchased from the Institute of Microbiology, the Chinese Academy of Science, China. The fungal species were maintained at 4 °C on potato dextrose agar slants that were transferred every 2 months.

### Isolation, identification and morphology of *L. theobromae*

The *L. theobromae* was isolated from a PAH contaminated soil collected from the Beijing Coking Plant in China^17^. First, 20 g (wet weight) of the soil was placed in a 250 mL wide-mouth bottle that contained 100 mL of MSM. The bottle was shaken overnight on a reciprocating shaker at 150 rpm and 28 °C. Next, the bottle was removed and remained static for 3 h. Five milliliters of the supernatant were transferred to a 100 mL wide-mouth bottle that contained 45 mL MSM, 25 mg L^−1^ BaP, 0.01 g mL^−1^ glucose, and 3% (*v*/*v*) methanol. This step was considered as the first enrichment run of the BaP-degrading fungi. During this process, 0.01 g mL^−1^ glucose and 3% (*v*/*v*) methanol were added to MSM to support fungal growth and to facilitate BaP dissolution, respectively. When fungal growth was visible, a second enrichment run was initiated by transferring 10% (*v*/*v*) of the the above culture into the same mixture of MSM, BaP, glucose, and methanol except that the concentration of BaP increased to 50 mg L^−1^. Then, the BaP concentration was gradually increased to 75 and 100 mg L^−1^ in the following enrichment runs. The last two runs did not contain glucose.

To isolate the fungi, the enriched culture was diluted by 10^7^ fold. Next, 0.1 mL of the sample was spread onto beef extract-peptone plates that were supplemented with cycloheximide (0.1 g L^−1^) and BaP (100 mg L^−1^) or onto potato-dextrose plates that were supplemented with penicillin G (60 g L^−1^), streptomycin sulfate (100 mg L^−1^), and BaP (100 mg L^−1^). After several incubation runs at 28 °C, fungal colonies were selected and re-plated on the same medium plates without antibiotics until pure colonies were obtained.

**Figure 1:**
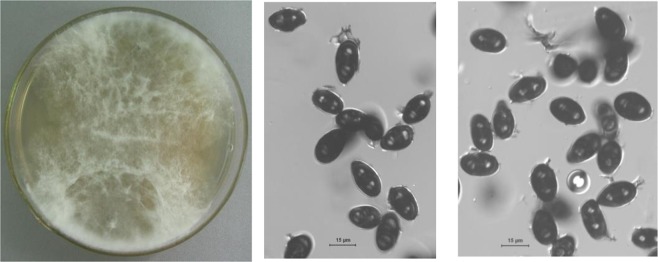
Morphological characterization of *L. theobromae*.

Because all of the single fungal colonies had similar macroscopic characteristics, representative colonies were selected for storage and were identified by the Institute of Microbiology, the Chinese Academy of Sciences, China. The fungus was identified by means of sequencing of the region ranging from the end of 18S rRNA gene to the beginning of 28S rRNA, encompassing complete ITS1, 5.8S rRNA and ITS2 region. It was identified as the *Lasiodiplodia theobromae* (GenBank sequence accession number KJ789100). The gene sequences of the *L. theobromae* were reported in the Supporting Information. The culturing colonial morphology of the *L. theobromae* indicated that the colony grew quickly on Potato Dextrose Agar (PDA), reaching a diameter of up to 65–70 mm in the dark after 3 d. The mycelia were white in the preliminary stage and became gray or black with time. The mycelia had a cotton-like texture and were sparse (Fig. 1). The front and back sides of the colony were the same color and had water-insoluble pigments. In addition, the microscopic morphology of the *L. theobromae* indicated that the mycelia were colorless, glossy, multi-branched, and separated, and had a width of 2.5–12.1 µm. A few black spots were formed during the later period and were identified as pycnidia. The conidium emerged from the orifices after maturity. The conidia were puce, oval or elliptical, straight, and amphicyte with a size of approximately 20.4–28.2 × 12.9–15.3 µm.

### Fungal inocula preparation

Fungal inocula were prepared by growing the separated fungal isolates on potato-dextrose plates at 32 °C until fungal mycelia appeared. The mycelia were transferred to potato-dextrose media and cultured for 2 d by shaking at 150 rpm under 28 °C. The flocculent mycelia were collected by centrifugation after washed twice with sterile MSM and suspended in an appropriate volume of MSM. This suspension was used as fungi inocula in downstream experiments. For the control groups, the fungal inocula were sterilized by pasteurizing at 121 °C for 30 min.

### BaP degradation by *L. theobromae* and *P. chrysosporium*

Specific volumes of the BaP stock solution and 3% methanol were added to 50 mL vials containing 20 mL of MSM to obtain an initial BaP concentration of 100 mg L^−1^. Appropriate volumes of the fungal inocula were added to the MSM-BaP culture to reach an initial population of 0.01 g (wet weight) mL^−1^. Control groups contained the same constituents except that the cells had been killed by pasteurization. All of the cultures were incubated by shaking on a rotary shaker operated at 150 rpm and 30 °C. Replicate samples were periodically sacrificed, the entire samples were used to measure the BaP concentrations, and the activities of LiP, MnP, and LAC.

Equal volumes of *L. theobromae* and *P. chrysosporium* inocula were separately added to the above test systems to compare the BaP degradation abilities and enzyme activities of the two fungi.

### Effects of metabolic substrates on BaP degradation and enzyme activities

The effects of metabolic substrates, including glucose, salicylic acid, and PHE, on BaP biodegradation and the activities of LiP and LAC were investigated. The glucose, salicylic acid, and PHE doses were 50, 200, and 300 mg L^−1^, respectively.

In addition, these metabolic substrates were added to the liquid culture media as a metabolic substrate for evaluating BaP biodegradation and the ligninolytic enzyme activities of *P. chrysosporium*, respectively.

### Effects of TW-80 on BaP degradation and enzyme activities

Specific amount of TW-80 was added to the culture media to obtain a concentration of 200 mg L^−1^ to observe its effects on BaP degradation and the secretion of LiP and LAC from the *L. theobromae*.

TW-80 was quantified following the method reported by Lu *et al*.^22^. Here, 1 mL of the culture was used to measure TW-80, and 2 mL of trichloromethane and 3 mL of ammonium cobalt thiocyanate were mixed with the culture, and then the mixtured was rotated at 2000 rpm for 30 min. The supernatant solution was removed from the mixture after delamination. The residual trichloromethane that contained TW-80 was detected at 620 nm with an ultraviolet-visible (UV-visible) spectrophotometer (Cary 50 Conc, varian, USA).

### BaP analysis

Twenty-five milliliter of liquid cultures was extracted twice with 25 mL of ethyl acetate by vigorous shaking. The two organic extracts were combined and concentrated by a rotary evaporator (<67 °C, 0.05 Mpa) to obtain a volume of 3 mL. Subsequently, the residue extract was concentrated until nearly dry under a gentle stream of high purity N_2_ gas. The recovery rate of the extraction was greater than 90%. Finally, samples were dissolved in 1.0 mL of methanol prior to HPLC (Agilent 1200, USA) analysis. HPLC analysis was performed by using a Venusil XBP C_18_ column (4.6 mm × 150 mm × 5 μm, 150 Å, Agela Technologies, China), and a methanol-water (90:10 *v*/*v*) solution was used as the mobile phase with a flow rate of 1.0 mL min^−1^. BaP was detected at 263 nm with an ultraviolet detector.

### Biomass and enzyme activity analysis

To evaluate the effect of 3% methanol on fungal biomass, the *L. theobromae*. mycelia were transferred to PDA and cultured for 2 d by shaking at 150 rpm under 28 °C. PDA cultures and PDA cultures added with 3% methanol were filtered using Whatman no. 1 paper, and mycelia were washed with 120 mL of deionized water and then dried to constant weight at 90 °C, respectively.

The entire liquid culture was frozen at −20 °C, thawed, and centrifuged to remove the mycelium and high molecular weight polysaccharide slime. Next, the supernatant was sequentially passed through a Whatman No. 1 filter, a 0.45 µm membrane filter and an Amicon YM10 membrane filter. Enzyme activities were monitored with a UV-visible spectrophotometer. LiP was assayed following the methods of Tien and Kirk^23^ by recording absorbance increases at 310 nm due to the oxidation of veratryl alcohol to veratryl aldehyde. The MnP was assayed in 0.1 mM MnSO_4_ and H_2_O_2_ with 0.01% phenol red as a substrate. Reactions were stopped by adding NaOH (5 mol L^−1^) before measuring the absorbance at 610 nm. The LAC activity was determined by measuring the increasing absorbance at 420 nm, which resulted from the oxidation of 0.5 mM ABTS in 0.1 M sodium acetate buffer (pH 5.0). One unit of enzyme activity was defined as the amount of enzyme that was required to oxidize 1 µmol of substrate min^−1^. Enzyme activity was expressed as U L^−1^ of culture filtrate. The results are presented as an average of triplicates with a standard error of <5%.

### BaP intermediate measurement and quantitative

2 mL culture was taken from the MSM culture containing BaP (10 mg/L) and *L. theobromae* (0.01 g wet weight/mL). Its pH was adjusted to 2.0 for acidification by 1 mol/L HCl, then dispersed and dissolved using 0.2 mL acetonitrile and 4 mL ethyl acetate, respectively. *L. theobromae* was removed by centrifugation. 1 mL of supernatant was taken and evaporated to dryness under high-purity N_2_ stream. Dried samples were derivatized in the mixture of hexane (100 μL) and Bis (trimethylsilyl) trifluroacetamide (BSTFA) (200 μL). The mixtures derivatized were heated at 60 °C for 12 h, and set to 2 mL using hexane and further analyzed with a Shimadzu Model 2010 gas chromatograph (GC) coupled with a Model QP2010 mass spectrometer (MS) (Shimadzu, Japan) using electronic ionization (EI) in the selected ion monitoring (SIM) mode. A TR-5MS (30 m × 0.25 mm i.d., 0.25 µm film thickness) capillary column was used to separate PAHs. The column temperature was initiated at 70 °C (held for 1 min) and increased to 260 °C at 10 °C/min (held for 2 min), then from 260 °C to 300 °C at 4 °C/min (held for 4 min). Auto injection of 1 µL of the samples was conducted in the splitless mode. Helium was used as the carrier gas at a flow rate of 1 mL/min. The ion source and interface temperatures were set to 250 °C and 280 °C, respectively. The temperature of MS detector was 180 °C and the mass scanning ranged from m/z 50 to m/z 550.

BaP intermediate quantification uses the initial concentration of BaP as a standard comparison, concentration of degradation products is equal to the ration of each product area and the area of BaP initial concentration multiply the initial concentration of BaP.

All experiments were conducted in triplicat. The results were used to calculate the means and standard deviations. When necessary, the results were analyzed with a one-way analysis of variance (ANOVA) test in SPSS 11.0.

## Results and Discussion

### BaP degradation by *L. theobromae* and its enzyme activities

**Figure 2:**
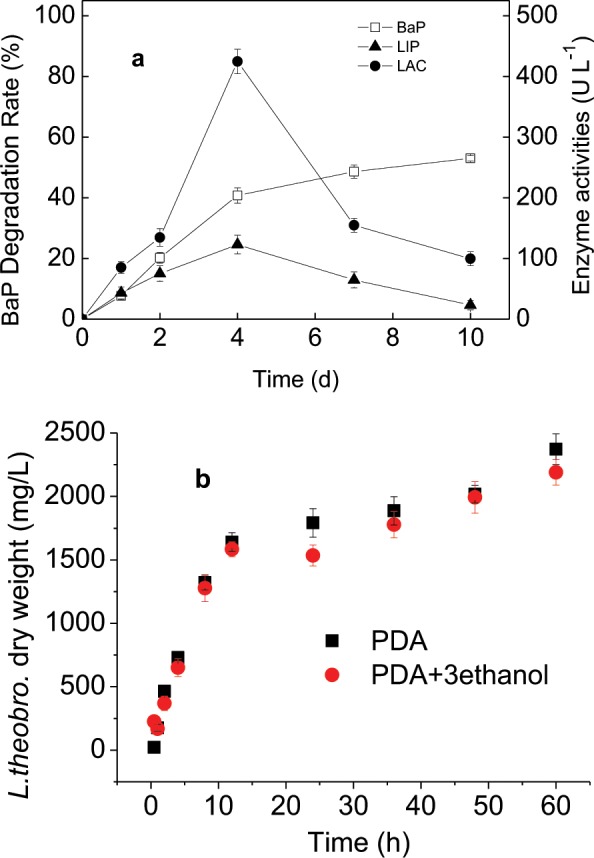
Kinetics of BaP degradation by the *L. theobromae* in MSM and enzyme activities during BaP biodegradation (**a**) and the effect of methanol on dry weight of L *L. theobromae* (**b**). Bars represent the standard errors for replicates (n = 3).

Figure 2(a) indicates that BaP degradation occurred rapidly during the early phase of incubation and then slowed. The degradation ratio reached 40.8 ± 2.5% on the 4^th^ day and 53.0 ± 0.9% after ten days of incubation. Additionally, the effect of 3% methanol on the biomass of the *L. theobromae* showed methanol did not increased its dry weight (Fig. 2(b)), which improved methanol did not provide the carbon resource for *L. theobromae*. Moreover, to evaluate whether the *L. theobromae* can efficiently degrade BaP as its sole carbon source, the effect of co-metabolic substrate on BaP biodegradation was conducted in the following in the Section 3.2.

The kinetics of the LiP, MnP, and LAC activities produced by the *L. theobromae* were measured during BaP degradation (Fig. 2(a)). MnP was not detected. The LiP and LAC activities increased during the initial period and decreased after peaking on the 4^th^ day. The changing enzyme activities agreed with the BaP degrading rates, which suggested that these enzymes were involved in BaP degradation. The highest LiP and LAC activities were 123.0 ± 15.2 U L^−1^ and 424.9 ± 20.2 U L^−1^, respectively. Throughout the experiment, the LiP activity was relatively lower than the LAC activity.

### Metabolic substrate effects on BaP degradation and enzyme activities

**Figure 3:**
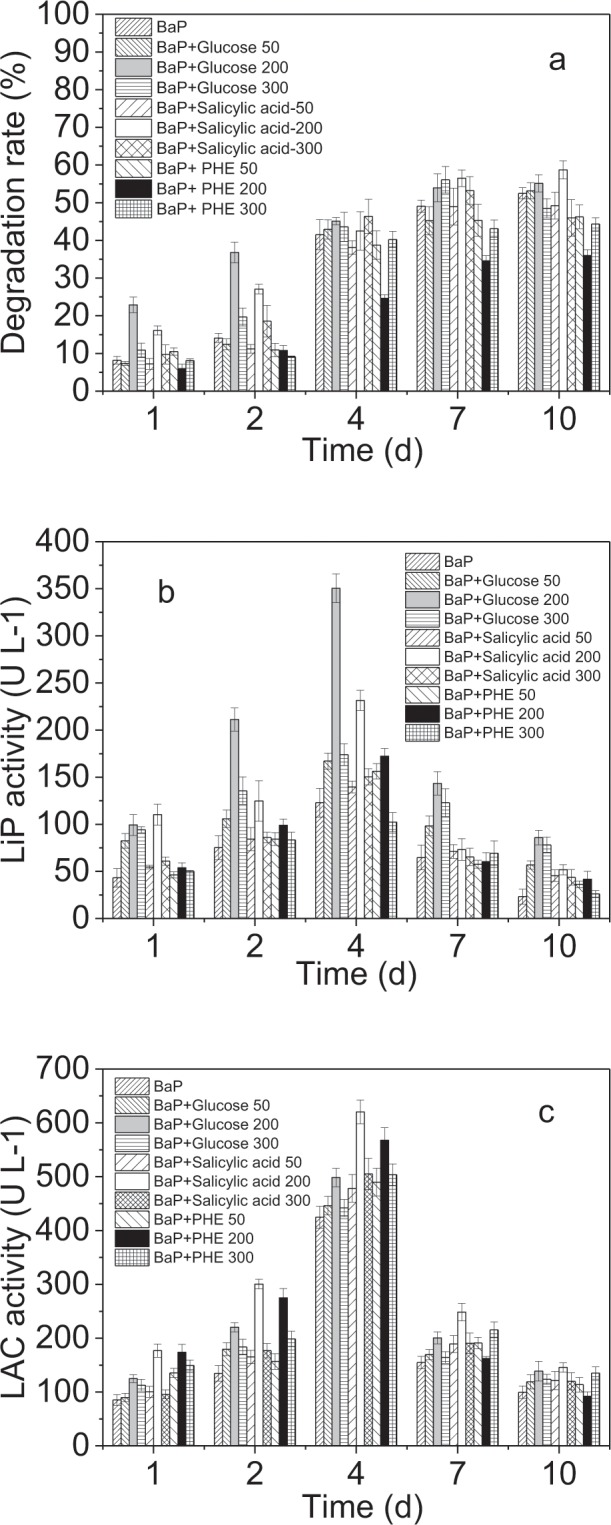
Effects of metabolic substrates on the BaP degradation and enzyme activities of the *L. theobromae*, including (**a**) BaP biodegradation; (**b**) LiP activity, and (**c**) LAC activity. Bars represent the standard errors for replicates (n = 3).

The addition of metabolic substrates is a common method for improving the biodegradation efficiency of recalcitrant compounds^3^. Figure 3 shows the effects of different concentrations of different metabolic substrates on BaP biodegradation by the *L. theobromaethe*. 200 mg/L glucose and salicylic acid slightly enhanced BaP biodegradation while PHE inhibited BaP biodegradation, while 50 and 300 mg/L metabolic substrates did not obviously influence. Take 200 mg/L metabolic substrate as an example, the final degradation ratio increased by 2.1% and 6.0% for groups following the addition of glucose and salicylic acid (*p* > 0.05), respectively, and decreased by 17.1% following the addition of PHE (*p* < 0.05). Although both PHE and salicylic acid contain benzene ring in their molecular structure, their properties are different. The PHE belongs to persistent organic pollutants, which has its own toxicity and its degradation intermediates is more toxicant^17^. However, salicylic acid is a monohydroxybenzoic acid with non-toxic, water solube, and completely biodegradable properties^5^. Thus, salicylic acid did not show inhibitory effect against BaP degradation. However, in the present study, metabolic substrates including glucose, salicylic acid, and PHE did not improve BaP degradation by the *L. theobromae*. In terms of the influences of metabolic substrates and methanol, we concluded that the *L. theobromae* can efficiently degrade BaP as its sole carbon source.

Figure 3(b,c) show the LiP and LAC activities in the presence of glucose, salicylic acid, and PHE. All the three substrates enhanced the LiP and LAC activities. The degree of enhancement for LiP occurred in the following order glucose > salicylic acid > PHE with peak values increased by 227.5 ± 8.2, 108.4 ± 6.7, and 49.5 ± 4.3 U L^−1^ (*p* < 0.05), respectively. In contrast, the enhancement in LAC activity by the three substrates occurred in the following order of salicylic acid > PHE > glucose with peak values increased by 195.6 ± 10.1, 142.8 ± 3.6 and 73.6 ± 4.4 U L^−1^ (*p* < 0.05), respectively. Therefore, these substrates enhanced the secretion of ligninolytic enzymes. Literatures noted that a growth substrate can initiate growth of the organism and to induce the production of catabolic enzymes^24,25^. For example, BaP degrading-enzymes can be induced by the low-molecular weight substrate phenanthrene and by salicylate to degrade the BaP^26^. And glucose is often used as a carbon source and energy source to enhance the strain growth. Thus, the increase of secretion of ligninolytic enzymes was attributed to the addition of extra metabolic substrates, which could increase the microorganism’s contact with its favorite substrates. And the favorite substrate could enhance the strain’s growth and its secretion of BaP degrading-enzymes^27^. Here, PHE increased the secretion of enzymes because PHE is a low molecular weight PAHs, which as a carbon source is more easily used than BaP by *L. theobromaethe*. Therefore, when BaP and PHE coexist, the carbon source increases, which promotes the LiP and LAC activity. However, the BaP degradation rate did not increase with the increasing enzyme concentration. This result was mainly attributed to the competition between the two substrates (Fig. S1). Figure 3(c) showed that glucose enhanced lowest LAC enzymes among other two substrates. Glucose enters to the carbon metabolism from the beginning of the glycolysis pathway. After entry into the cell, glucose is converted to G6P by hexokinase (HK) to prevent efflux from the cell^28^. Most of the glucose is used for the generation of two molecules of lactate that this might be regarded as a waste of energy. We assume that lactate produced by glucose uptake resulted in the LAC activity lower than other enzymes.

### Effects of TW-80 on BaP degradation and enzyme activities

**Figure 4:**
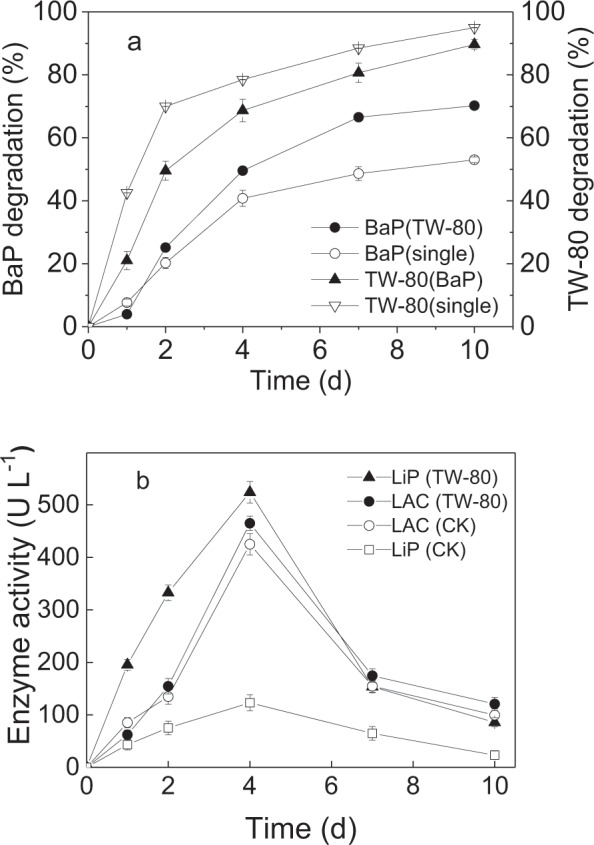
Effects of TW-80 on BaP degradation (**a**,**b**) effect of TW-80 on the LiP and LAC activities of the *L. theobromae* during BaP degradation. (Note: ∇ represents TW-80 degradation, ▲ represents TW-80 degradation in the coexistence system of TW-80 and BaP, ○ represents BaP degradation, ● represents BaP degradation in the coexistence system of TW-80 and BaP.) Bars represent the standard errors for replicates (n = 3).

Figure 4(a) shows the effects of the TW-80 surfactant on BaP degradation by the *L. theobromae*. TW-80 enhanced the final BaP biodegradation ratio by 17.2 ± 2.4% (*p* < 0.05), because solubility of BaP was increased due to the formation of micelle^7,29^. Here, we observed that the TW-80 content decreased significantly during BaP biodegradation. When TW-80 was added as the sole carbon source, TW-80 was completely degraded by the *L. theobromae* (Fig. 4(a)). Kotterman *et al*.^30^ observed that TW-80 was gradually metabolized by *Bjerkandera sp*. (strain BOS55) when it was added as the surfactant to enhance the solubility of anthracene. In addition, Zang *et al*.^31^ found that *Bacillus*-07 could use TW-80 for energy without inhibiting its growth. When BaP and TW-80 coexist, although the degradation rate of TW-80 is decreased, it can still be completely degraded (Fig. 4(a)). The complete degradation of TW-80 by the *L. theobromae* is advantageous for applying the *L. theobromae* in the bioremediation of a contaminated field since this can prevent the secondary pollution by TW-80.

Figure 4(b) shows that TW-80 enhanced the LiP and LAC activities, especially for LiP. The peak LiP activity could reach 523.9 ± 20.5 U L^−1^ in the TW80 and BaP system, which is approximately four times greater than the value (122.98 U L^−1^) of the system without TW-80. The LAC activity reached 464.9 ± 13.7 U L^−1^ following the addition of TW-80, which was 40.0 ± 3.1 U L^−1^ greater than the original value with 424.87 U L^−1^ (*p* < 0.05). Similar results were observed by Shome *et al*.^32^ who reported that nonionic surfactants (Brij-30, Brij-92, Tween-20, and Tween-80) could enhance lipase activity from *Chromobacterium Viscosum* (CV-lipase). In our previous study, we found that TW-80 could enhance the population of the *L. theobromae* because TW-80 provided a carbon source for the *L. theobromae*^30^. Hence, TW-80 as a surfactant can enhance solubility and bioavailability of BaP, thus it can improve the degradation of BaP. Moreover, TW-80 enhanced the secretion of LAC enzyme and this also improved the degradation of BaP.

### Role of LiP and LAC during BaP degradation

In this study, some interesting phenomena regarding the capability of the *L. theobromae* ligninolytic enzymes for degrading BaP were observed. There are two types of ligninolytic enzymes: peroxidases (LiP and MnP) and laccases (LAC). These enzymes are secreted extracellularly, compete for the same substrates, catalyze radical formation by oxidation to attack of molecule and destabilize bonds in a molecule, the radicals formed interact with each other or with the fungal metabolites and generate other radicals that continue the process^33–35^. The four substrates (i.e., glucose, salicylic acid, PHE, and TW-80) all enhanced the secretion of LiP and LAC from the *L. theobromae*. The peak LiP concentrations increased by 179, 87, 40, and 327% in the presence of glucose, salicylic acid, PHE, and TW-80, respectively, (*p* < 0.05). In contrast, the LAC concentrations increased by 18, 46, 34, and 9%, respectively. Glucose and TW-80 resulted in relatively higher LiP concentrations and lower LAC concentrations. In contrast, salicylic acid and PHE resulted in relatively higher LAC concentrations and lower LiP concentrations. The salicylic acid and PHE contain benzene rings, while benzene ring does not exist in the molecular structure of glucose and TW-80. The metabolic substrate used for the cometabolism of chemicals with similar structures had a greater capacity because it can induce the release of specific enzymes for degrading the target organics^36^. Thus, we assumed that metabolic substrates that contain benzene rings resulted in greater LAC concentrations that could enhance the activity of LAC to cleave the benzene ring compared to LiP. Several studies regarding the PAH biodegradation have noted that MnP and LAC are the most important enzymes for PAH oxidation^37,38^. The classical action of laccases are oxidation of substrate by transferring electrons to oxygen in one-electron steps results in the polymerization of phenols and/or the forming of quinones^39^. Crude laccase preparations were able to oxidize both anthracene and the potent carcinogen BaP^40^. However, discussions regarding LiP biodegradation are rare. Here, our results showed that LiP was more easily induced than LAC by glucose and TW-80. Glucose is a monosaccharide with the following chemical formula H-(C=O)–(CHOH)_5_-H. The five hydroxyl groups in glucose are arranged in a specific way along its six-carbon backbone that are connected by common carbon-carbon bonds. The main component of TW-80 is unsaturated fatty acid. Steffen *et al*.^2^ reported that LiP is potentially generated during the degradation of TW-80 when LiP is involved in lipid peroxidation. In addition, Pozdnyakova *et al*.^37^ reported that LiP is a relatively nonspecific enzyme that is known to be able to oxidize phenolic aromatic substrates, a variety of nonphenolic lignin model compounds, and a range of organic compounds. LiP is unique because it can oxidizes an aromatic ring to a cationic radical by taking electrons from the benzene ring, and then undergoes a cleavage reaction, resulting in the oxidative cleavage of carbon-carbon and ether bonds (C-O-C) in nonphenolic aromatic substrates with a high redox potential^41^. However, the role of LiP in PAH degradation is limited to a narrow range of compounds based on their ionization potential (IP) values. Thus, LiP is relatively nonspecific and may be easily induced by organics with simple structures due to its nonspecificity (glucose and TW-80 in this case).

Thus, LAC played a more significant role during BaP degradation by the *L. theobromae* than LiP. In addition, LiP showed relatively no specificity for its substrates.

### Comparison between *L. theobromae* and *P. chrysosporium*

In this study, the appearance of the *L. theobromae* was similar to that of *P. chrysosporium*, which produces white mycelium and secretes lignin-degrading enzymes during growth^42^. *P. chrysosporium* has been widely studied for PAH degradation due to its high efficiency^36,43^. Thus, because the *L. theobromae* is a novel strain for BaP biodegradation, it is important to compare the BaP degradation ability and enzyme activities of the *L. theobromae* and *P. chrysosporium*.

**Figure 5:**
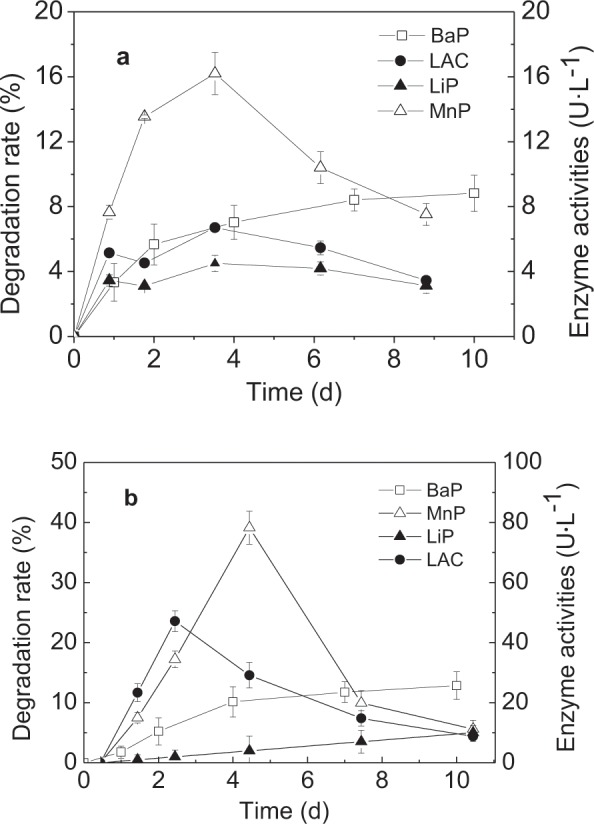
Kinetics of BaP degradation and the enzyme activities of *P. chrysosporium* in MSM (**a**) and MSM with the addition of salicylic acid (**b**). Bars represent the standard errors for replicates (n = 3).

BaP was not significantly degraded by *P. chrysosporium* in the MSM, the degradation rate was less than 10% (Fig. 5(a)). However, the degradation ratio of BaP by the *L. theobromae* in the MSM was 53% after ten days of incubation (Fig. 2). To enhance BaP biodegradation, salicylic acid was added as a metabolic substrate and BaP degradation by *P. chrysosporium* increased to 13% (Fig. 5(b)). In addition, BaP degradation by the *L. theobromae* remained the same. Therefore, BaP cannot be solely degraded by the *P. chrysosporium*, however, the *L. theobromae* could use it as single carbon source.

The time-dependent ligninolytic activities were measured during BaP biodegradation by *P. chrysosporium* (Fig. 5(a)). The LiP, MnP, and LAC activities were 4.5 ± 0.5, 16.2 ± 1.3 and 6.7 ± 0.2 U L^−1^ in the MSM with addition with BaP, respectively (Fig. 5a). In addition, the peak LiP, MnP, and LAC activities of the *L. theobromae* were 123.0 ± 15.2, non-detectable, and 424.9 ± 20.2 U L^−1^ when BaP was the sole carbon source (Fig. 2(a)). When salicylic acid was added, the enzyme activities from *P. chrysosporium* (Fig. 5(b)) and the *L. theobromae* increased (Fig. 3(b,c)). For example, the LiP, MnP, and LAC activities from *P. chrysosporium* increased by up to 47.2 ± 2.8, 78.3 ± 4.9, and 82.9 ± 4.3 U L^−1^, respectively, while the LiP and LAC activities from the *L. theobromae* reached 231.4 ± 10.8 and 620.4 ± 22.1 U L^−1^ (*p* < 0.05), respectively. Therefore, the enzymes produced by the *P. chrysosporium* and *L. theobromae* during BaP biodegradation were different. The *L. theobromae* produced greater LiP and LAC enzyme concentrations than *P. chrysosporium*. In general, the *L. theobromae* is a promising species for PAH degradation.

Additionally, Figs. 2–5 show that the activities of LiP, MnP, and LAC increased firstly and then decreased with prolonged platforms of BaP-biodegradation. Because BaP is more easily utilized by the *L. theobromae* as a source of carbon and energy in the initial stage of BaP degradation, and this was more likely to promote enzyme production. However, with degradation time prolonged, a series of BaP intermediate products were produced simultaneously^10^. These intermediate products may be harmful to the *L. theobromae*, which may result in that degrading-enzymes were reduced gradually. Therefore, activities of LiP, MnP, and LAC increased firstly and then decreased with prolonged platforms of BaP-biodegradation.

### BaP biodegradation intermediates

**Figure 6:**
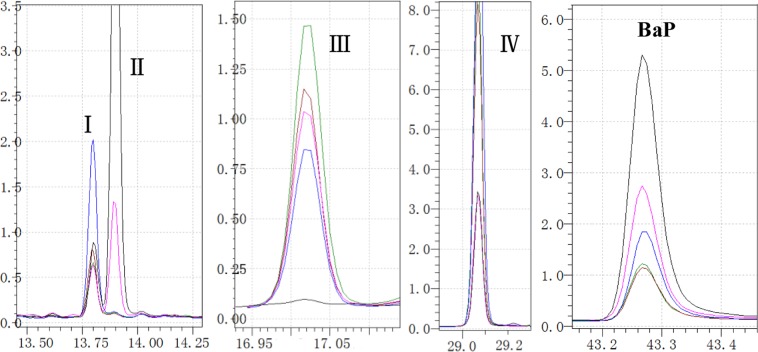
Production of unidentified compounds formed during 10 mg/L of benzo[a]pyrene by *L.theobromae* in MSM culture.

**Figure 7:**
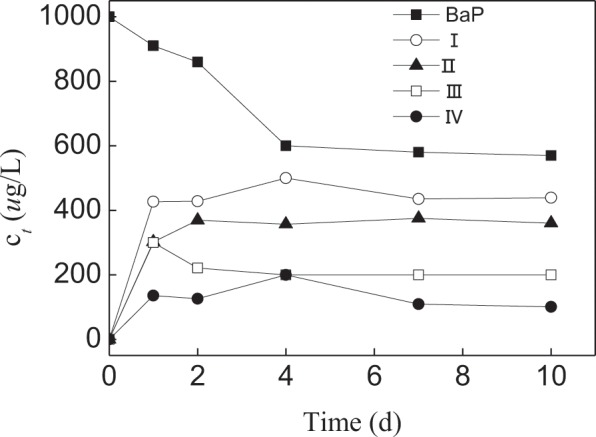
Time course of compounds formed during 10 mg/L of benzo[a]pyrene degradation by *L.theobromae* in MSM culture.

The intermediates formed in the BaP biodegradation process were monitored using GC-MS analysis. Except for the peak of BaP, four metabolites (compounds I, II, III, and IV) were observed during benzo[a]pyrene degradation by *L. theobromae* (Fig. 6). Though these degradation products were not identified, Figs. 6 and 7 showed that BaP concentration were significantly lower after the 4-day *L. theobromae* incubated, in contrast, four metabolite concentrations first increased and then decreased or obtained the platform with prolonged BaP biodegradation time. The literature noted that white-rot fungi that produce ligninolytic enzymes can oxidise PAHs by generating free radicals (ie hydroxyl free radicals) by the donation of one electron, which oxidises the PAH ring. This generates a selection of PAH-quinones and acids rather than dihydrodiols^44^. And then decompose PAHs into CO_2_ and H_2_O through hydrogenation, dehydration and so on. For example, BaP 1,6-, 3, 6- and/or 6,12-quinones have been detected as BaP polar metabolites^45–47^, and found the mineralisation of BaP by microorganisim after growth on glucose or by crude and purified extracellular ligninase preparations^48–50^. The authors proposed that the mechanism of *L. theobromae* for BaP was likely to similar with that of white-rot fungi.

## Conclusions

The present study was to elucidate the enzymatic mechanisms of BaP degradation by the *L. theobromae* that was isolated from the Beijing Coking Plant in China. The results showed that BaP as its sole carbon source was degraded up to 53% by the *L. theobromae* after ten days. Among three ligninolytic enzymes including LiP, MnP, and LAC, LAC played a more important role in BaP biodegradation than LiP, however, MnP enzyme was not detected. The metabolic substrates such as glucose and salicylic acid slightly enhanced BaP biodegradation, while PHE inhibited degradation. These metabolic substrates enhanced LiP and LAC secretions. The surfactant of TW-80 obviously enhanced BaP degradation ratio by up to 70% and enzyme activities. Additionally, for the degradation capacity and enzyme activities, the *L. theobromae* was more efficient than the widely studied *P. chrysosporium* species. Therefore, the study indicates a favorable potential for using the *L. theobromae* as a new strain for removing PAHs from contaminated sites.

## Supplementary information

Supplemental Material.
