# Supplementary material for: Enzyme activities during Benzo[a]pyrene degradation by the fungus Lasiodiplodia theobromae isolated from a polluted soil
Source: Sci Rep. 2020 Jan 21;10:865. doi: 10.1038/s41598-020-57692-6 (PMC6972742; doi:10.1038/s41598-020-57692-6)
Supplement: Supplementary file 1 — Supplemental Material. [file 41598_2020_57692_MOESM1_ESM.docx]

**Supplementary Material**

**Enzyme activities during Benzo[a]pyrene degradation by the fungus *Lasiodiplodia theobromae* isolated from a polluted soil**

**Huimin Cao, Cuiping Wang*, Haibin Liu, Weili Jia, Hongwen Sun***

MOE Key Laboratory of Pollution Processes and Environmental Criteria, College of Environmental Science and Engineering, Nankai University, Tianjin 300071, People’s Republic of China

* Corresponding author

Mailing address: College of Environmental Science and Engineering, Nankai University, Tianjin 300071, People’s Republic of China

Phone: +86-22-2359241; Fax: +86-22-23509241.

Email: [sunhongwen@nankai.edu.cn](mailto:sunhongwen@nankai.edu.cn); wangcp@nankai.edu.cn

Identification of isolated fungal strain was performed by means of sequencing of region ranging from the end of 18S rRNA gene to the beginning of 28S rRNA, encompassing complete ITS1, 5.8S rRNA and ITS2 region. A sequence of 542 bp is listed below:

TCCGTAGGTGAACCTGCGGAAGGATCATTACCGAGTTTTCGAGCTCCGGCTCGACTCTCCCACCCTTTGTGAACGTACCTCTGTTGCTTTGGCGGCTCCGGCCGCCAAAGGACCTTCAAACTCCAGTCAGTAAACGCAGACGTCTGATAAACAAGTTAATAAACTAAAACTTTCAACAACGGATCTCTTGGTTCTGGCATCGATGAAGAACGCAGCGAAATGCGATAAGTAATGTGAATTGCAGAATTCAGTGAATCATCGAATCTTTGAACGCACATTGCGCCCCTTGGTATTCCGGGGGGCATGCCTGTTCGAGCGTCATTACAACCCTCAAGCTCTGCTTGGAATTGGGCACCGTCCTCACTGCGGACGCGCCTCAAAGACCTCGGCGGTGGCTGTTCAGCCCTCAAGCGTAGTAGAATACACCTCGCTTTGGAGCGGTTGGCGTCGCCCGCCGGACGAACCTTCTGAACTTTTCTCAAGGTTGACCTCGGATCAGGTAGGGATACCCGCTGAACTTAAGCATATCAATAAGCGGAGGA

The above 18S rRNA and 28S rRNA gene sequences from this fungus was compared with the available database (Genbank) using the BLAST program at the National Center for Biotechnology Information (NCBI, http: // [www.ncbi.nlm.nih.gov/). The](http://www.ncbi.nlm.nih.gov/).%20The) fungus was identified as *Lasiodiplodia theobromae*.

**Fig. S1.** Degradation rates of BaP and PHE by *L. theobromae* under BaP and PHE single and mixed. (note: ▲ represents BaP degradation in the coexistence system of PHE and BaP. △ represents BaP degradation. ● represents PHE degradation in the coexistence system of PHE and BaP. ○ represents PHE degradation.)
